# The anteroposterior axis of the tibia is approximately perpendicular to the anterior pelvic plane in the standing position in healthy Japanese subjects

**DOI:** 10.1186/s13018-017-0642-8

**Published:** 2017-09-25

**Authors:** Norio Imai, Dai Miyasaka, Tomoyuki Ito, Hayato Suzuki, Izumi Minato, Naoto Endo

**Affiliations:** 1 Division of Comprehensive Geriatrics in Community, Niigata University Graduate School of Medical and Dental Sciences, Niigata, 9518510 Japan; 2 Department of Orthopedic Surgery, Niigata University Medical and Dental Hospital, Niigata, 9518510 Japan; 3 Department of Orthopedic Surgery, Saiseikai Niigata Daini Hospital, Niigata, 9501104 Japan; 4 Department of Orthopedic Surgery, Niigata Rinko Hospital, Niigata, 9508725 Japan

**Keywords:** Anteroposterior axis of tibia, Femoral neck anteversion, Clinical epicondylar axis, Anterior pelvic plane, three-dimensional assessment, Lower extremity alignment

## Abstract

**Background:**

We previously reported that the clinical epicondylar axis (CEA) was approximately parallel to the transverse axis of the anterior pelvic plane (APP) in the standing position in normal subjects. The purpose of this study was to investigate the rotational alignment between APP in the standing position and the anteroposterior (AP) axis of the tibia relative to pelvic coordination in normal subjects.

**Methods:**

This study included 68 healthy Japanese, 24 males and 44 females, without lumbago and knee pain. Femoral neck anteversion (FNA), condylar twist angle, and knee rotation angle were measured in femoral coordination. The angle between the femoral neck axis and CEA transverse axis of APP was also measured, and the angle between the AP axis of the tibia and the transverse axis of APP was calculated. The mean value of knee rotation angle was 0.23° and 2.06° in male and female subjects, respectively.

**Results:**

There was a moderate positive correlation between FNA and the femoral axis angle relative to the transverse axis of APP. The knee rotation angle relative to APP was 0.33° and 1.56° in male and female subjects, respectively, and the tibia AP axis was approximately perpendicular to the transverse axis of APP in the standing position. Regarding validation, we obtained high interclass correlation coefficients for both intraobserver and interobserver reliability.

**Conclusion:**

We found that the knee rotation angle was almost 0° and that the tibia AP axis was approximately perpendicular to the CEA. The tibia AP axis was also approximately perpendicular to the transverse axis of the APP in standing position.

## Background

Alignment of the lower extremity is determined by the spatial and geometrical relationship between the femur and tibia. It is important to assess the alignment of the lower extremity in order to determine and identify diseases with abnormal alignment in the lower extremities, such as osteoarthritis of the knee and hip, patellofemoral disorder, patellar dislocation, and congenital malalignment [1–7]. Previously, lower extremity alignment was usually assessed with two-dimensional (2D) plain radiography, using the femorotibial angle or hip-knee-ankle angle in the coronal plane alone [7–9]. However, measurements with 2D radiography are influenced by the direction of the radiation source and the orientation of the pelvis and lower extremities of subjects [10]. Therefore, it is considered insufficient for precise measurements with regard to its accuracy and reproducibility. Moreover, rotational alignment cannot be evaluated with plain radiography.

Several studies have reported that the anteroposterior (AP) axis of the tibia, which is defined by a line passing through the middle of the posterior cruciate ligament and the medial border of the patellar tendon attachment (Akagi’s line), is almost perpendicular to the clinical epicondylar axis (CEA) [11–13].

We previously reported that the CEA runs approximately parallel to the transverse axis of the anterior pelvic plane (APP) in the standing position in healthy subjects [14]. However, to our knowledge, no report has described the rotational alignment between APP and AP axis of the tibia, and if they are perpendicular to CEA in the standing position.

The purpose of this study was to investigate the rotational alignment between APP in the standing position and AP axis of the tibia relative to pelvic coordination in normal subjects.

## Methods

### Subjects

For this study, we included 68 healthy Japanese (24 males, mean age, 51.7 ± 10.5 years; 44 females, mean age, 54.3 ± 11.0 years) without lumbago and knee pain and without any abnormal findings of the knee and spine on radiographic examination. They were enrolled from the family of outpatients and medical staff; therefore, they are all middle age people. This study was performed with the approval of the institutional research board of Niigata University Medical and Dental Hospital, and written informed consent was obtained from all participants. Radiographic examinations, including biplanar computed radiography images, were performed in the standing position, where each subject adopted a relaxed position with their knees fully extended and the toes aligned to the shoulders. Computed tomography was also performed in the supine position with the knees fully extended.

### Measurements

**Fig. 1:**
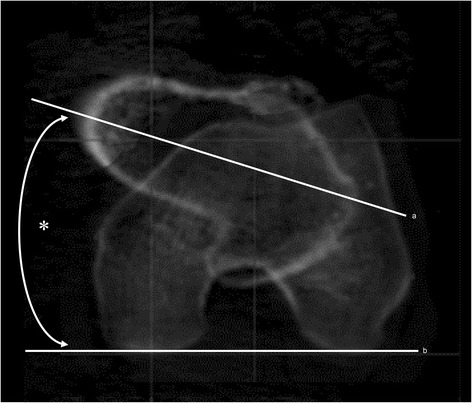
Measurement of FNA. FNA (✻) is the angle between the femoral neck axis (a) and the PCA (b). FNA: femoral neck anteversion, PCA: positive external rotation

**Fig. 2:**
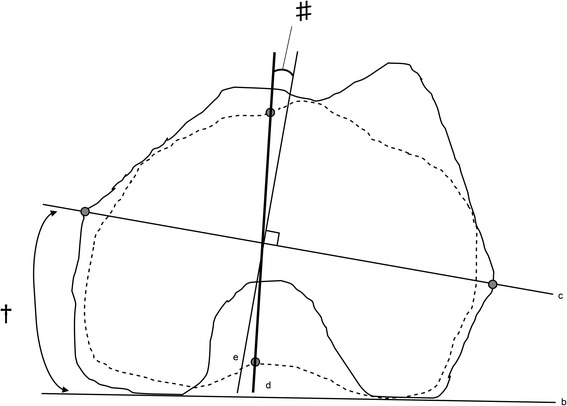
Measurement of CTA and knee rotation angle. CTA (†) is the angle between the PCA (b) and the CEA (c). KRA (#) is the angle between the tibial AP axis [11] (d) and the line perpendicular to the femoral CEA (e). The solid lines represent the contour of the projected femoral condyle onto the femoral horizontal plane. The dotted lines represent the contour of the projected tibial condyle onto the femoral horizontal plane. CEA: clinical epicondylar axis, CTA: condylar twist angle, KRA: knee rotation angle, PCA: positive external rotation

We used ZedHip® software (Lexi, Tokyo, Japan) to create three-dimensional (3D) digital bone models of the pelvis and femur and to accurately reconstruct the spatial relationship between them [12, 15, 16]. We adjusted the 3D pelvis model to the APP [17], which contains both the anterior superior iliac spines and the pubic symphysis, the origin of this pelvis coordinate system. In the ZedHip system, when the pelvis was adjusted to the APP, other bones such as the femur and tibia synchronously moved according to the pelvis position. With regard to the femoral coordinate system, the 3D model of the femur was positioned with the retrocondylar plane, which contains the bilateral posterior condyles and the most prominent posterior point of the greater trochanter [18]. The femoral neck axis was defined according to the method by Sugano et al. [19], as the tangent to the anterior and posterior margin of the femoral neck in the plane just below the femoral head. Femoral neck anteversion (FNA) was measured as the angle between the femoral neck axis as above and the posterior condylar axis (PCA) (Fig. 1). Further, the CEA was defined as the line connecting the most prominent point of the medial epicondyle and the lateral epicondylar prominence. The condylar twist angle (CTA) was measured as the angle connecting the CEA and PCA (positive values means CEA is externally rotated relative to PCA) (Fig. 2). The determination of CEA and the measurement of CTA were also performed in the retrocondylar plane. The line through the midpoint of the lateral epicondylar prominence and the most prominent point of the medial epicondyle, and perpendicular to the CEA was defined as the femoral AP axis. With regard to the AP axis of the tibia, Akagi’s line [11] was selected. The knee rotation angle (KRA) was measured as the angle connecting the femoral AP axis and the AP axis of the tibia, projected onto the horizontal plane of the femoral coordinate system (Fig. 2). In this present study, negative values were defined as the internal rotation of the tibia relative to the femur, and positive values as the external rotation.

**Fig. 3:**
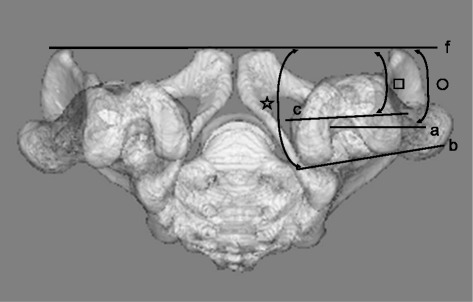
Measurement of APP-FNA, APP-PCA, and APP-CEA. APP-FNA (〇), APP-CEA (□), and APP-PCA (☆) were defined as the angles connecting the FNA (**a**), PCA (**b**), and CEA (**c**), respectively, to transverse axis APP (**f**). APP: anterior pelvic plane, CEA: clinical epicondylar axis, FNA: femoral neck anteversion, PCA: positive external rotation

FNA, CEA, and PCA relative to APP were also measured in the standing position (APP-FNA, APP-CEA, and APP-PCA) using HipCAS® software (Lexi, Tokyo, Japan). The 3D digital bone models were projected onto the biplanar computed radiography images to match the contours of the 3D digital models with the computed radiography images for rotations and translations [12, 15, 16]. Kobayashi et al. [16] previously described the accuracy of HipCAS® in creating a 3D digital bone model that accurately reproduced the spatial relationship between the pelvis and the femur, and calculating the various alignment parameters within 1° and 1 mm of accuracy. Therefore, projection error and misalignment were estimated to be small in the current study. APP-FNA, APP-CEA, and APP-PCA were the angles that connected the FNA, CEA, and PCA projected onto the transverse plane of the pelvis and APP was the line connecting both anterior superior iliac spines (Fig. 3).

**Fig. 4:**
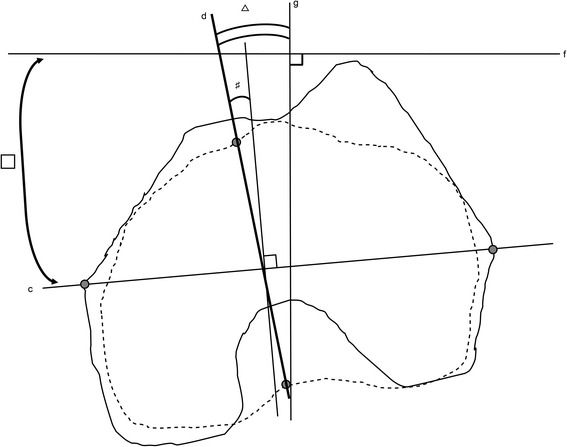
Calculation of the estimated AP axis of the tibia and APP transverse axis (f). We calculated the estimated angle between the AP axis of the tibia (d) and the perpendicular line to APP (g) from KRA (#) and APP-CEA (□) with the formula; (AP axis of the tibia relative to APP transverse axis △) = (APP-CEA □) minus (KRA #). The solid lines represent the contour of the projected femoral condyle onto the femoral horizontal plane. The dotted lines represent the contour of the projected tibial condyle onto the femoral horizontal plane. AP: anteroposterior, APP: anterior pelvic plane, CEA: clinical epicondylar axis (c), KRA: knee rotation angle

Lastly, we calculated the estimated angle between APP and the AP axis of the tibia from KRA in femoral coordination and APP-CEA with the formula: (AP axis of the tibia relative to the APP transverse axis) = (APP-CEA) − (KRA) (Fig. 4).

### Statistical analysis

We used SPSS statistical software (SPSS version 24, Inc., Chicago, IL, USA) to analyze the data. Regarding FNA, CTA, KRA, APP-FNA, and APP-CEA, we used Pearson coefficients to determine the correlation coefficients. To evaluate variation, we calculated the mean absolute difference (MAD), variability (standard deviation), and intraobserver reliabilities with intraclass correlation coefficients (ICCs) and a two-sided 95% confidential interval. We measured intraobserver reliability with twice measurements by one observer at least 1-week intervals. Moreover, we also compared the measurements to assess the interobserver reliability with single measurement of two observers. A *p* value < 0.01 was considered statistically significant.

## Results

The details of the participants are shown in Table 1. There was a moderate positive correlation between FNA and APP-FNA (regression equation; *y* = 0.43× + 3.64) and a negative correlation between FNA and APP-CEA (regression equation; *y* = −0.22× + 2.70) (Table 2). This finding indicated a trend towards greater FNA leading to more internal rotation, while the participants with greater FNA had greater APP-FNA compared to those with less FNA.

**Table 1:** Details of the participants

	Male (*n* = 24)	Female (*n* = 44)
Age (years)	51.7 ± 10.5	54.3 ± 11.0
Body height (cm)	167.4 ± 6.4	152.8 ± 5.7
Body weight (kg)	63.7 ± 9.4	52.0 ± 7.3
BMI	22.7 ± 2.5	22.3 ± 2.8

Values are mean ± standard deviation

*BMI* body mass index

**Table 2:** Correlation coefficient between each parameter in the participants

	FNA (deg)	CTA (deg)	KRA (deg)	APP-FNA (deg)	APP-CEA (deg)	APP-tibia AP axis (deg)
FNA (deg)		0.175	0.071	0.578*	−0.346*	−0.083
		0.075	0.113	0.410*	−0.337*	−0.007
CTA (deg)			0.167	0.068	0.174	0.041
			−0.223	0.128	0.185	0.080
KRA (deg)				−0.223	−0.245	−0.021
				0.050	−0.246	−0.236
APP-FNA (deg)					−0.281*	0.177
					−0.377*	−0.056
APP-CEA (deg)						0.144
						0.013

Upper row: male, Lower row: female

*AP* anteroposterior, *APP*: anterior pelvic plane, *APP-CEA* clinical epicondylar axis relative to APP, *APP-FNA* FNA relative to APP, *CTA* condylar twist angle, *FNA* femoral neck anteversion, *KRA* knee rotation angle

*p < 0.01

KRA relative to APP was 0.33° in men and 1.56° in women (Table 3). This finding meant that the tibia AP axis was approximately perpendicular to the APP in the standing position.

**Table 3:** Measurement of anatomical and positional angles

	Male (*n* = 24)	Female (*n* = 44)
FNA (deg)	12.42 ± 10.19	17.17 ± 9.26
CTA (deg)	7.17 ± 1.99	7.27 ± 1.86
KRA (deg)	0.23 ± 4.46	2.06 ± 6.91
APP-FNA (deg)	9.50 ± 11.66	10.93 ± 8.10
APP-CEA (deg)	0.10 ± 3.60	−0.50 ± 4.11
APP-tibia AP axis (deg)	0.33 ± 4.03	1.56 ± 5.51

Values are mean ± standard deviation

*AP* anteroposterior, *APP* anterior pelvic plane, *APP-CEA* clinical epicondylar axis relative to APP, *APP-FNA* FNA relative to APP, *CTA* condylar twist angle, *FNA* femoral neck anteversion, *KRA* knee rotation angle

Regarding validation, we obtained a high ICC for both intraobserver and interobserver reliability (Table 4).

**Table 4:** Intraobserver reliabilities

	Intraobserver reliability		Interobserver reliability	
	MAD ± SD	ICC	MAD ± SD	ICC
FNA (deg)	1.31 ± 1.48	0.904	1.58 ± 1.78	0.854
CTA (deg)	0.63 ± 0.47	0.937	0.78 ± 0.56	0.921
KRA(deg)	1.45 ± 1.68	0.868	1.88 ± 1.87	0.824
APP-FNA (deg)	1.58 ± 1.88	0.812	1.79 ± 1.95	0.801
APP-CEA (deg)	0.74 ± 0.54	0.934	0.86 ± 0.78	0.912
APP-tibia AP axis (deg)	1.21 ± 0.88	0.886	1.57 ± 1.30	0.862

*AP* anteroposterior, *APP* anterior pelvic plane, *APP-CEA* clinical epicondylar axis relative to APP, *APP-FNA* FNA relative to APP, *CTA* condylar twist angle, *FNA* femoral neck anteversion, *ICC* interclass correlation coefficient, *KRA* knee rotation angle, *MAD* mean absolute difference, *SD* standard deviation

## Discussion

In the current study, we evaluated the spatial relationship between the tibia AP axis and APP in normal subjects.

We found that the mean value of KRA was 0.23 ± 4.46° in men and 2.06 ± 6.91° in women. These values are similar to that obtained in previous reports [11–13]. Moreover, KRA relative to APP was 0.33° in men and 1.56° in women. These findings mean that the tibia AP axis is almost perpendicular to APP in the standing position. However, to our knowledge, no other study has considered the spatial relationship of these axes. Therefore, to the best of our knowledge, this is the first study to establish the spatial relationship between the tibia AP axis and APP, and determine that they are at almost a right angle in the standing position.

It is generally known that the pelvis is kinesiologically almost symmetrical and that its movement is symmetrical: in flexion and extension relative to the sagittal plane [20]; in internal and external rotation relative to the horizontal plane [21, 22]; and in abduction and adduction relative to the coronal plane [21, 22], during normal gait. Consequently, the transverse axis of the APP is regarded as one of the functional axes, not only of the pelvis but also of the hip joint. CEA has also been considered as the functional flexion-extension axis of the knee [23, 24].

We previously described that CEA was approximately parallel to the transverse axis of the APP plane in the standing position in normal subjects [14] and in our current study, we reported that the KRA was almost 0° and that the tibia AP axis was approximately perpendicular to the CEA. This new concept in the current study may be important to integrate these two axes with regard to not only the anatomical reference, but also the kinesiology of the lower extremity including the pelvis, hip joint, and knee joint. These findings may prove helpful to decide the alignment of implants in total hip or knee arthroplasty, treatment for patellar dislocation, and positional alignment investigation such as gait analysis**.**

The current study has several limitations. First, only a few subjects and only middle age people were enrolled. Therefore, we cannot perform any power study. Second, KRA was examined in the supine position, while APP-FNA and APP-CEA were examined in the standing position. However, according to several reports, the difference in KRA between the supine and standing positions seems negligible [12, 13]. Kozanek et al. stated that the KRA was approximately 3° at contralateral toe-off, and nearly 0° from ipsilateral heel-rise to contralateral heel-strike, respectively, during the stance phase of treadmill gait [25]. Third, there was a difference in the plane of the measurement; the KRA was examined in femoral coordination, while APP-FNA and APP-CEA were examined in pelvis coordination. Chen et al. reported that the tibia was internally rotated approximately 3° relative to the femur when the knee was flexed from 0° to 8° [26]. We preliminarily measured the femur in the 5° flexion position and 3° adducted position relative to APP in the standing position on average and our computer simulation found that with the lower extremity in this position, the expected difference of the angle was not more than 0.5°. Therefore, we believe that the position of the lower extremity did not affect the results of this study. Fourth, these anatomical and positional angles such as FNA and KRA vary among patients. It should be noted that these angles are not constant.

The strong point of the current study was measurement by using a 3D digital bone model. Previously, it may be considered insufficient for precise measurements with regard to its accuracy and reproducibility; furthermore, rotational alignment cannot be evaluated with plain radiography. However, we used HipCAS® in this study, which its accurate reproducibility of the spatial relationship between the pelvis and the femur was previously described, and calculate the various alignment parameters within 1° and 1 mm of accuracy; therefore, projection error and misalignment was estimated to be small in the current study.

## Conclusions

We found that the KRA was almost 0° and that the tibia AP axis was approximately perpendicular to the CEA. The tibia AP axis was also found to be approximately perpendicular to the transverse axis of the APP in the standing position. Our findings seemed rational considering not only the neutral position of the hip and knee joints but also the anatomical and kinesiological validation of our results in normal subjects. This new concept presented in the current study may be important for the integration of these three axes: the transverse axis of APP, CEA, and tibia AP axis, not regarding the anatomical reference alone, but also the kinesiology of the pelvis, hip joint, and knee joint.

## Abbreviations

AP: Anteroposterior
APP: Anterior pelvic plane
CEA: Clinical epicondylar axis
CTA: Condylar twist angle
FNA: Femoral neck anteversion
ICC: Intraclass correlation coefficient
KRA: Knee rotation angle
MAD: Mean absolute difference
PCA: Posterior condylar axis
